# Ultrasound detection of placenta accreta in the first trimester of pregnancy

**Published:** 2014-06

**Authors:** Fatemeh Rahimi-Sharbaf, Ashraf Jamal, Elaheh Mesdaghinia, Masoumeh Abedzadeh- Kalahroudi, Shirin Niroomanesh, Fatemeh Atoof

**Affiliations:** 1 *Perinatalogy Devision, Department of Obstetrics and Gynecology, Faculty of Medicine, Tehran University of Medical Sciences, Tehran, Iran.*; 2 *Trauma Nursing Research Center, Kashan University of Medical Sciences, Kashan, Iran.*; 3 *Trauma Research Center, Kashan University of Medical Sciences, Kashan, Iran.*

**Keywords:** *Placenta accreta*, *Ultrasonography*, *Screening*, *Sensitivity*, *Specificity*

## Abstract

**Background:** Placenta accreta is considered a life-threatening condition and the main cause of maternal mortality. Prenatal diagnosis of placenta accreta usually is made by clinical presentation, imaging studies like ultrasound and MRI in the second and third trimester.

**Objective:** To determine accuracy of ultrasound findings for placenta accreta in the first trimester of pregnancy.

**Materials and Methods: **In a longitudinal study 323 high risk patients for placenta accreta were assessed. The eligible women were examined by vaginal and abdominal ultrasound for gestational sac and placental localization and they were followed up until the end of pregnancy. The ultrasound findings were compared with histopathological examinations as a gold standard. The sensitivity, specificity, positive and negative predictive value of ultrasound were estimated for the first trimester and compared with other 2 trimesters in the case of repeated ultrasound examination.

**Results: **Ultrasound examinations in the first trimester revealed that 28 cases had the findings in favor of placenta accreta which ultimately was confirmed in 7 cases. The ultrasound sensitivity and specificity for detecting placenta accreta in the first trimester was 41% [95% CI: 16.2-62.7] and 88% [95% CI: 88.2-94.6] respectively.

**Conclusion:** Ultrasound screening for placenta accreta in the first trimester of pregnancy could not achieve the high sensitivity as second and third trimester of pregnancy.

## Introduction

Placenta accreta indicates deep attachment of the placenta to myometrium due to the absence of decidua basalis (1). In this condition, at least some or all parts of the placenta may not be removed causing postpartum hemorrhage (2, 3). The incidence rate is varied between 1/110 to 1/2500 deliveries (4-6). It has been increasing up to 4 times due to the increased Cesarean section rate (6). Placenta accreta mostly represents as the placenta previa in the third trimester having the incidence rate of 9.3%, while in 0.005% of cases the placenta has a normal position (7). 

Placenta accreta is considered a life-threatening condition and the main cause of maternal mortality, postpartum hysterectomy, admission to ICU, and an inadvertent laceration to intestine or bladder during cesarean section (8, 9). The incidence rate may be increased in some conditions such as repeated Cesarean section, placenta previa, previous uterine surgery especially if the placenta embeds at the site of previous incision scar, maternal age over 35 years old, smoking habit, past history of surgery like myomectomy and curettage (7, 10-12). Prenatal diagnosis usually is made by clinical presentation, imaging studies like ultrasound and MRI in the second and third trimester (13-15). 

Ultrasound findings in the first trimester include low lying gestational sac, hypo echoic placental regions, irregular placental- myometrial interface, and placenta previa (16). Generally, a low lying gestational sac in the first trimester indicates the implantation of trophoblast at the site of previous uterine scar (17). The major ultrasound findings in the second and third trimester consist of hypo echoic placental regions, and placental lacunae (18). 

The sensitivity of ultrasound findings varies in different studies and is reported between 33-100% and the specificity is also widely different (11, 13-15, 19). The value of ultrasound for detecting placenta accreta in high risk population has been investigated. The sensitivity has been reported 77-99% and the predictive value of 65-93% (13, 15, 20). Usually, placenta accreta is diagnosed in the third trimester with severe hemorrhage during curettage (21). The recent studies have made the prenatal diagnosis in the weeks of 11-14 (22). Some case studies have elaborated placenta accreta signs at the early stage of the first trimester when it is difficult to distinguish placenta accreta from ectopic pregnancy situated at the lower uterine segment of previous Cesarean section (17, 23, 24). The objective of the current study is to determine the accuracy of ultrasound for detecting placenta accrete in the first trimester in women with history of repeated cesarean section or other uterine surgeries.

## Materials and methods

This is a longitudinal study of 323 high risk women for placenta accreta who attended antenatal clinics at three university hospital during 2011-2012. Convenience sampling was used for patient's selection. Approval was obtained by the perinatology group Tehran University of Medical Sciences and Informed consent was obtained from all participants. 

Inclusion criteria were history of abortion, Dilatation and Curettage (D&C), Cesarean section (C/S) and other uterine surgeries such as myomectomy. The eligible women with gestational age of 9-14 weeks were examined by vaginal and abdominal Doppler ultrasound in the first trimester for gestational sac implantation, placenta localization, placental myometrial interface and interplacental lakes. 

They were followed up by ultrasound examinations in the second and third trimester for ultrasound findings of placental site, hypoechoic placental regions, irregular interface between myometrium and placenta, and increased vascularity between myometrium and bladder. Ultrasound examinations were done in the second trimester at 16-24 weeks of pregnancy and in 3^rd^ trimester at 30-34 weeks of pregnancy. Maternal characteristics including maternal age, gravidity, parity, history of abortion, Dilatation and Curettage (D&C), Cesarean section (C/S) and other uterine surgeries were recorded. 

All cases were followed for outcome till term. The definite diagnosis of placenta accreta was based on histopathological examination as a gold standard and ultrasound findings were compared with these results. All ultrasound examinations were performed by perinatology fellows using Ziemens Ultrasound machine and abdominal and vaginal transducer (3133 and 8189 MHZ) respectively. Also pathological examinations were performed by pathologists in three centers.

**Statistical analysis**

The statistical analysis was performed using SPSS software. The sensitivity, specificity, positive and negative predictive values of ultrasound was estimated for each trimester. Chi-square and T-test were used to investigate the association between underlying variables and placenta accreta prevalence rate. 

## Results

The mean (median) for maternal age, gravidity and parity were 30.80 (30), 2.9 (3), and 1.3 (1); respectively. There was a significant difference between the women having placenta accreta and those without placenta accreta regarding the parity, abortion, and previous cesarean section. Overall, out of 323 studied women, placenta accreta was diagnosed in 17 cases (5.3%) based on histopathological findings during pregnancy period. In 9 patients pathological examinations didn’t perform due to spontaneous abortion. Serial ultrasound could not detect placenta accreta in 4 cases (18%) during pregnancy (case No 1, 10, 14, 16). Out of 17 confirmed cases of placenta accreta, 9 cases (53%) were accompanied with placenta previa. 

**Figure 1:**
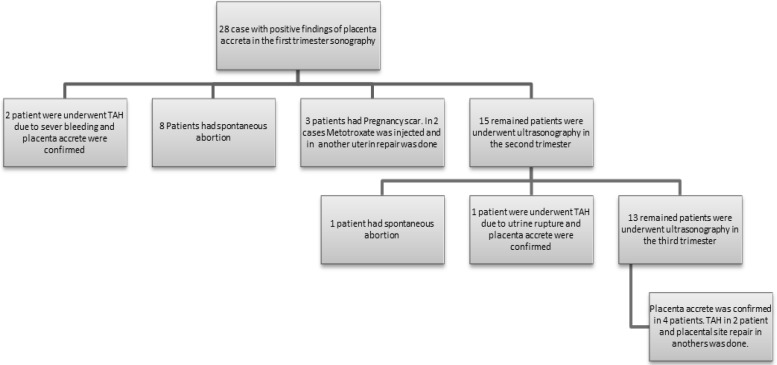
Outcome of 28 women with positive findings of placenta accrete in the first trimester ultrasonography

Table I shows ultrasound characteristics of studied patients. Ultrasound examinations in the first trimester revealed that 28 cases had the findings in favor of placenta accreta (low lying gestational sac or placenta at the site of previous uterine scar) which ultimately was confirmed in 7 cases (Figure 1). Findings demonstrate that the ultrasound sensitivity and specificity to detect placenta accreta in the first trimester were 41% and 88%; respectively. Considering the detected placenta previa and accreta cases in the second trimester, the sensitivity and specificity were reported 60% and 83.5%; correspondingly. Moreover; given the detected placenta previa and accreta cases in the third trimester, the sensitivity and specificity were reported 71.4% and 88.5%: consecutively (Table II).

**Table I:** Characteristics of women with confirmed placenta accrete diagnosis

**Case**	**Age**	**Cesarean number**	**Ultrasond 1** ^st^ ** trimester**	**Ultrasond 2** ^nd^ ** trimester**	**Ultrasond 3 ** ^rd ^ **trimester**	**Outcome**
1	42	4	Upper gestational sac Placenta anterior	Placenta anterior	Placenta anterior	C/S ^1^& TAH^ 2^
2	35	3	Incisional placenta	Placenta previa	Placenta previa and accreta	C/S & TAH
3	29	3	Low gestational sac Incisional placenta	-	-	D&C^3^ -TAH
4	32	3	Low gestational sac Incisional placenta	Placenta previa	Placenta previa and accreta	Preterm labor -C/S & TAH
5	31	1	Placenta previa	Placenta previa	-	V/B^4^ -Uterine rupture & TAH
6	29	0	Upper gestational sac Placenta anterior	Placenta accreta	Placenta accreta	C/S & TAH
7	28	1	Low gestational sac Incisional placenta	Placenta previa and accreta	Placenta previa and accreta	C/S & placental site repair
8	25	1	Upper gestational sac Placenta anterior	Placenta previa and accreta	Placenta previa and accreta	Preterm labor -C/S & TAH
9	35	2	Upper gestational sac Incisional placenta	Placenta previa	Placenta previa and accreta	C/S & placental site repair
10	29	3	Upper gestational sac Placenta posterior	Placenta posterior	Placenta posterior	C/S & TAH
11	29	2	Upper gestational sac Placenta anterior	Placenta previa	Placenta previa and accreta	C/S & TAH
12	40	2	Upper gestational sac Placenta anterior	Placenta previa	Placenta previa and accreta	C/S & TAH
13	33	1	Upper gestational sac Placenta anterior	Placenta anterior	Placenta previa and accreta	C/S & TAH
14	42	3	Upper gestational sac Placenta anterior	Placenta anterior	Placenta anterior	C/S & TAH
15	34	2	Upper gestational sac Placenta anterior	Placenta previa	Placenta previa and accreta	C/S & TAH Maternal Death
16	31	1	Upper gestational sac Placenta posterior	Placenta posterior	Placenta posterior	C/S & placental site repair
17	35	4	low gestational sac Placenta previa	-	-	D&C - TAH

1-Cesarean Section

2- Total Abdominal Hysterectomy

3- Dilatation and Curretage

4- Vaginal Bleeding

**Table II:** Sensitivity and specificity of ultrasound for placenta accrete diagnosis according to different pregnancy trimester

**Ultrasonography**	**Sensitivity** **(CI** * ** 95%)**	**Specificity** **(CI 95%)**	**Positive predictive value** **(CI 95%)**	**Negative predictive value** **(CI 95%)**
First trimester	41% (16.2-62.7)	88% (88.2-94.6)	16% (8.2-38.5)	96% (93.4 -98.1)
Second trimester	60% (32.3-83.7)	83.5%(78.8-87.5)	15.5% (7.35-27.4)	97.6 %(94.9 -99.1)
Third trimester	71.4% (41.9-91.6)	88.5% (84.3-91.9)	22.7% (11.5 -37.8)	98.5% (96.2-99.6)

* Confidence Interval

## Discussion

The findings demonstrated that out of 17 detected placenta accreta cases by pathology, ultrasound were true positive in 7 cases at first trimester, so ultrasound in the first trimester could not detect placenta accreta in 10 cases. Therefore, the ultrasound sensitivity and specificity for detecting placenta accreta in the first trimester in this study were estimated 41% and 88%: respectively. There are limited studies conducted in the literature for screening of ultrasound in the first trimester and there are some case studies in which ultrasound diagnostic value has still been unclear at this stage of pregnancy. It is not clear whether implated trophoblast on the previous uterine scar be a determinant factor for developing placenta accreta. 

As a study conducted by Miller *et al *showed that placenta accreta may be implanted at a further distance of uterine scars (7). The study also revealed that out of 15 detected placenta accreta cases by ultrasound in the second trimester and confirmed with pathologic examination, 9 cases were true positive and ultrasound failed to detect it in 6 cases. Given the detected placenta previa and accreta, the ultrasound sensitivity and specificity were 60% and 83.5% respectively in the second trimester. Additionally, the findings demonstrated that out of 14 detected placenta accreta cases by ultrasound in the third trimester, 10 cases were true positive and ultrasound failed to detect it in 4 cases. So given the detected placenta previa and accreta, the ultrasound sensitivity and specificity reached to 71.4% and 88.5%: consecutively with accuracy of 87%. 

Most studies regarding the ultrasound accuracy were fulfilled in the second and third trimester with different sensitivity and specificity. In a cohort study by Lim *et al* 30 high risk pregnant women were evaluated by ultrasound which placenta accreta was confirmed in 9 cases. They reported 67% sensitivity and 50% specificity (23). Another study by Dwyer *et al* conducted on 32 patients compared the accuracy of abdominal ultrasound with MRI for detecting placenta accreta which was confirmed in 15 cases at the delivery time. Out of 15 cases, placenta accreta was detected in 14 ones so sensitivity was 93% (CI: 80-100%). 

Also ultrasound rejected placenta accreta in 12 cases out of 17 ones. Therefore, the specificity was 71% (CI: 49-93%) (24). Differences between this study and our findings could be due to research method (historical cohort in Dwyer study) and sampling, frequency of the abdominal transducer used (a higher frequency will improve resolution) and sonographer experiences. A research accomplished on 65188 Malaysian pregnant women during the years 1996- 2005 showed 40 placenta accreta cases out of which 77.5% were associated with placenta previa. 35% of them had at least history of one cesarean section and 25% one curettage. In this series 31 cases underwent ultrasound examination during pregnancy. Out of them, ultrasound detected placenta accreta in 26 cases (83.6%). 

The mean gestational age at the time of detection was 28.3 weeks and the earliest time was 19^th^ week of gestation (1). Another study by Woording *et al* screened 12 suspected patients at 25th week of gestation of which 92% had the past history of placenta previa. Hysterectomy was performed for 83% of them due to placenta accrete and 2 cases had false positive report of placenta accreta which ultimately were placenta previa (25). The obtained results from the mentioned studies had better sensitivity that is explained by the type of ultrasound (vaginal or abdominal), sonographer’s experience, gestational age and quality of image resolution. 

In addition, the ultrasound accuracy can be affected by introducing abdominal transducer or bladder content (26, 27). Overall, the results showed that 82% of placenta accreta cases were detected before labor. So, this information provides a team for making consult and also planned delivery that ia accompanied with better outcome. Some advantages that can be mentioned are as follows: selecting delivery time, decision making for removing the placenta following labor and using prophylactic regimens or alternative therapies such as ligating internal iliac artery during operation, arterial embolization, and administrating methotrexate after labor.

## Conclusion

In conclusion although ultrasound has good accuracy for placenta accreta in the second and third trimester, but it did not achieve high accuracy in the first trimester. It seems focusing in this field can help to reduce maternal mortality and morbidity. 
